# An Auditory Illusion of Proximity of the Source Induced by Sonic Crystals

**DOI:** 10.1371/journal.pone.0133271

**Published:** 2015-07-29

**Authors:** Ignacio Spiousas, Pablo E. Etchemendy, Ramiro O. Vergara, Esteban R. Calcagno, Manuel C. Eguia

**Affiliations:** Laboratorio de Acústica y Percepción Sonora, Universidad Nacional de Quilmes, Bernal, Buenos Aires, Argentina; Kyoto University, JAPAN

## Abstract

In this work we report an illusion of proximity of a sound source created by a sonic crystal placed between the source and a listener. This effect seems, at first, paradoxical to naïve listeners since the sonic crystal is an obstacle formed by almost densely packed cylindrical scatterers. Even when the singular acoustical properties of these periodic composite materials have been studied extensively (including band gaps, deaf bands, negative refraction, and birrefringence), the possible perceptual effects remain unexplored. The illusion reported here is studied through acoustical measurements and a psychophysical experiment. The results of the acoustical measurements showed that, for a certain frequency range and region in space where the focusing phenomenon takes place, the sonic crystal induces substantial increases in binaural intensity, direct-to-reverberant energy ratio and interaural cross-correlation values, all cues involved in the auditory perception of distance. Consistently, the results of the psychophysical experiment revealed that the presence of the sonic crystal between the sound source and the listener produces a significant reduction of the perceived relative distance to the sound source.

## Introduction

The task of reconstructing the auditory space from the pressure waves arriving at the eardrums is one of the most challenging operations that our brains can perform. We have learned to localize and segregate sound sources integrating several temporal and spectral cues, and comparing them between our ears [1]. Also, we are able to detect and localize sources even in the presence of reverberation (precedence effect [2]), and competing sounds (cocktail party effect [3]). However, under certain circumstances, our brains can be tricked, and the localization and spatial features of the sources can be manipulated without changing the actual sources of sound. An echo from a distant wall, a reflection from a curved surface or the formation of a “creeping wave” along a wall (as happens in the whispering gallery effect [4, 5]), dramatically alters our spatial perception of the sound sources. In this last case, the speech from a distant talker might be heard as being emitted from a nearby location or even from inside our heads.

Auditory illusions are scarcely investigated but, as many other illusions, they can provide insights into how the brain organizes perceptual information. Moreover, the vast majority of the illusions studied are related to temporal [6] and spectral attributes of sound, and only very few to spatial effects. Among the spatial effects studied are the precedence effect [2], the saltation effect [7] and the “in-head” localization illusion [8] (that produces an effect similar to that observed in the whispering galleries).

On the other hand, spatial auditory illusions have been present since ancient times in building acoustics through the (sometimes intentional) utilization of curved walls and resonances in closed spaces. Examples of this are some greek amphiteaters [9] and early christian churches [10], and ancient galleries and courts [11–13]. However, these provide static and highly idiosyncratic examples of spatial auditory illusions. Therefore, one can ask if these illusions can be studied in a more systematic way. Here, we report and study an auditory spatial illusion that takes place in a real acoustic space, but that can also be controlled in a laboratory environment: the changes in the apparent distance to a sound source that is located behind a sonic crystal. Sonic crystals are essentially periodic structures that can block, shape and manipulate the propagation of sound [14]. Preliminary observations in our laboratory revealed that sonic crystals can also modify the apparent localization of acoustics sources, and subjective reports pointed to the existence of an “in-head” effect similar to those produced by curved walls.

### Sonic Crystals

**Fig 1:**
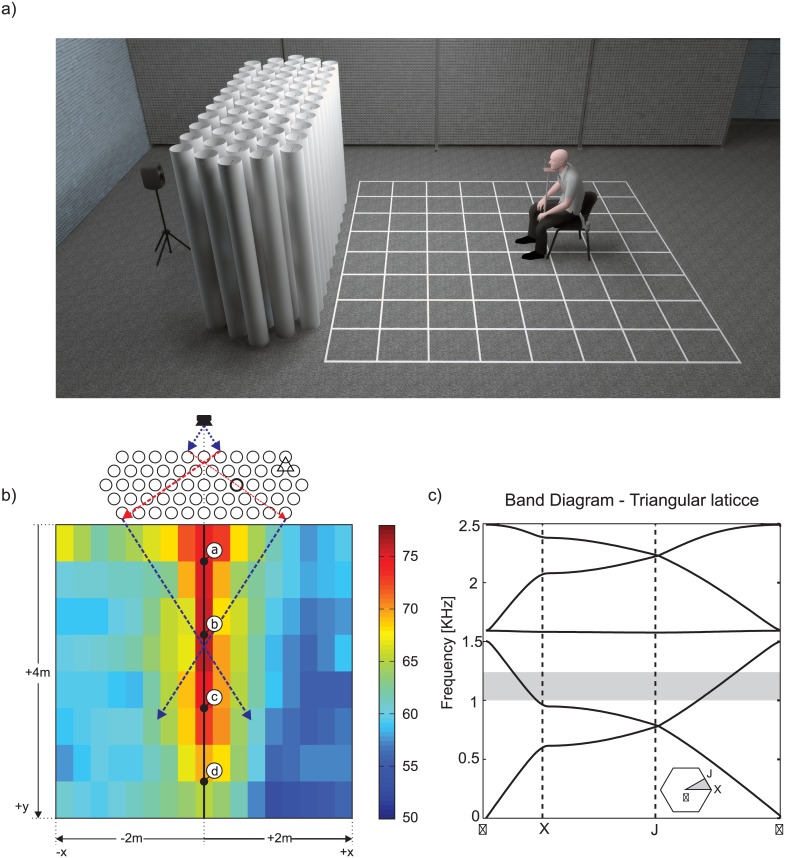
Experimental configuration employed in the binaural recordings and band diagram of the sonic crystal slab used on the experiments. **(a)** Realistic 3D rendering of the sonic crystal slab, loudspeaker and one of the listener positions employed in the binaural recordings. **(b)** Schematic representation of the experimental setup and sound intensity map (for a noise band with central frequency 1.12 kHz, 1/3 octave bandwidth). The sound source position is shown by a loudspeaker icon on the top of the figure. For the recordings, the dummy head and the experimental subjects were placed on the black dots along the central line (a-d). The red and blue rays correspond to a geometric acoustics representation of the focusing by negative refraction. **(c)** Band diagram corresponding to the sonic crystal slab used in the experiments. The gray area on the band diagram delimitates a negative refraction band (central frequency 1.12 kHz, 1/3 octave bandwidth). The monaural intensity of this noise band was measured using a sound meter level and it is plotted as a colormap on panel (b). The insets is the first reduced Brillouin zone of the reciprocal space showing the points *X*, *J* and Γ that defines the directions of the wave vector in the reciprocal space (e.g. the Γ−*X* direction corresponds to the normal incidence in the direct space).

The first evidence of the unusual acoustical properties of a sonic crystal came from the measurements performed on a minimalist sculpture, designed by Eusebio Sempere and exhibited at the Juan March Foundation in Madrid [14]. This seminal work showed that the repetition of rigid cylinder rods arranged in a lattice, inhibited the sound transmission for certain frequency ranges (band-gaps), in an analogous way to the already known effect of photonic crystals on light [15]. In its simplest realization, a sonic crystal (SC) consists in an bi-dimensional array of rigid cylinders in air. As an example, the SC slab employed in this work is displayed in Fig 1a. The particularity of this kind of composite material that make it interesting for studying perceptual effects is that it displays a large variation of its acoustical properties only by changing its geometrical configuration [16].

Sonic crystals have been extensively studied due to their singular transmission and reflection properties. These properties extend from acoustic band gaps [17] to negative refraction [18], negative bi-refraction [19] sound focusing [20] and imaging [21]. When a sound wave impinges a sonic crystal slab the resulting behavior dramatically changes depending on its frequency: it can be blocked (in the band-gap frequency range), refracted, diffracted, splitted or focused. The phenomenon of focusing by negative refraction [22, 23] is illustrated in Fig 1b, where we display a schematic view of the experimental setup and the recorded sound intensity for a narrow-band filtered noise within the focusing frequency range. It can be seen that the sonic crystal, even when formed by almost densely packed cylinders, instead of attenuating the sound intensity, can act as an acoustic lens, focusing the sound. This happens for a certain range of frequencies and for a limited region in space, that can be altered by changing the geometrical parameters of the sonic crystal (for example, the distance between cylinders). In this way, sonic crystals are useful devices for controlling the propagation of sound. Even when the unusual acoustical properties of SCs were thoroughly studied, the possible perceptual effects induced by these type of materials remain unexplored. In this work, we investigate the illusion of proximity of the source created by the sonic crystal in the focusing region.

### Auditory Distance Perception (ADP)

The human auditory system extracts distance information from a number of perceptual cues that have different degrees of importance depending on the characteristics of the stimulus, the acoustic environment, and the experience of the listener [24]. The most studied cues are: sound intensity, direct-to-reverberant energy ratio, spectral and binaural cues.

Intensity is a primary cue for ADP [25]. In an anechoic or free field environment the relationship between the intensity and the distance between the sound source and the listener obeys an inverse-square law, which implies a 6 dB intensity loss for each doubling of distance. This relationship does not apply in reverberant environments where the variation of the overall stimulus intensity caused by changes in distance from the source is lower, as a result of the addition of reflected sound components [26]. Another cue for auditory distance is the differential change in the spectral content of wide-band auditory stimuli induced by the absorption properties of air [27]. When a broadband sound travels from the sound source to the listener it loses more energy in the high frequency region of the spectrum, making distant sources sound less “bright” that near ones. A common feature of these two cues is that they are relative, because prior information about the power and spectrum of the source is needed for estimating its distance [28]

Previous works showed that judgments of apparent source distance are more accurate in a reverberant than in an anechoic environment [29–31]. Several authors have suggested that this effect is caused by an absolute auditory cue of distance: the direct-to-reverberant energy ratio. In reverberant environments the intensity of the direct sound obeys the inverse square law, whereas the energy in the later arriving reflected portion remains relatively constant. This is an interesting cue because it does not depend on the intensity of the signal and can be effective in any environment where reflections occur (see [24, 32] for a review).

Binaural cues arise from the relative changes produced in the signals arriving at the left and right ears as the distance from the source is modified, and can be caused both by the direct sound and the reverberation. In the free field, changes in the binaural cues induced by changes in the egocentric distance are produced only when the sound source is located in the near field and out of the median plane. In this cases, the incident sound wave cannot be considered flat and its curvature depends on the distance between the source and the listener’s ears; this leads to an increase in the shadowing effect when the source is approaching the listener’s head, and therefore an increase in the interaural intensity difference between the ears. However, in reverberant environments, reflections alter the attributes of sound at the two ears differentially. Many of these changes depend on the distance between the listener and the sound source and thus have been proposed as ADP cues. For example, results obtained by Bronkhorst and Houtgast [33] show that ADP depends on the number of reflecting surfaces in the room and, in particular, on the number of reflecting lateral walls. In a later work, Bronkhorst concludes that the reverberation is a binaural cue and that it is based on interaural time differences between incoming reflected sounds [34]. In the same context, results obtained by Lokki et al [35] and Lokki and Patynen [36] have shown that early lateral reflections affect the ADP. The results of these studies show that lateral early reflections lead to the perception of closer and stronger sound than median plane reflections. Lateral early reflections produce larger interaural cross-correlation (IACC) differences than median plane reflections and it has been hypothesized that listeners can use this information as a cue for assess the distance to the sound source [37]. However, most of the evidence on this topic is indirect and the role of the IACC in ADP is not clear and it remains as an open question for future research.

### Experiment

The aim of the current study was to evaluate the significance of the auditory illusion of proximity of the source created by the sonic crystal and to investigate the role of the different cues implicated in this phenomenon. To this end, we performed acoustical measurements using a dummy-head and a psychophysical experiment with binaural recordings. In both cases the stimuli were bandpass filtered noise bursts and the recordings were made in a semi-reverberant room at different distances, with and without the sonic crystal placed between the source and the recording position. We selected three acoustical cues that are implicated in the auditory perception of distance: the binaural intensity (BI), the direct-to-reverberant energy ratio (DRR), and the inter-aural cross-correlation (IACC), and we modified the binaural recordings in order to suppress two of these cues for evaluating the effect of one at a time.

## Results

### Acoustical Cues

A realistic illustration of the experimental setup using the sonic crystal and a schematic diagram of the source and receiver positions are shown on Fig 1a and 1b, respectively. The recordings were made using a fixed sound source and placing a binaural dummy-head at perpendicular distances from the slab between 0.8 and 3.8 m, with steps of 1 m (black circles a-d in Fig 1b), using the midpoint between ears as a reference. The elevation of the dummy-head and the loudspeaker were both 1.2 m. The sound samples used for the recordings consisted of thirteen one-third-octave noise bands with central frequency ranging from 0.5 to 2 kHz with a one-sixth-octave step. All recordings were performed with and without the SC slab (see Materials and Methods for more detail), giving a total of 104 recorded samples (13 filtered noise bands × 4 positions × 2 SC-conditions).

Also, in Fig 1b we display the sound intensity map, measured with a sound level meter, corresponding to a one-third octave noise band centered in 1.12 kHz. It can be seen that there is a clear focusing effect: the sound energy is concentrated in a narrow region aligned with the speaker. This phenomenon is know as negative refraction focusing [18]. A simple way to understand the principle behind this negative refraction focusing phenomenon is to give it a geometric acoustics interpretation: sound rays emanating from the source are inverted along the tangent direction when entering into the sonic crystal, and inverted back when leaving. Thus, the energy emitted by a point source can be concentrated on the central line forming an image of the source, as depicted in Fig 1b with two representative sound rays.

A band diagram of the sonic crystal slab used for the recordings is shown in Fig 1c. The band diagram portrays the dispersion relation (the relation between the temporal frequency *ω* and the wave-vector *k*) for a plane wave propagating across the crystal and can be used to predict negative refraction bands. As an example, the same one-third octave noise band used in Fig 1a is displayed as a shaded area over the band diagram. Within this region, it can be noted that the wavelength of the wave (the inverse of the wave-vector modulus) becomes greater as the frequency rise, implying that the effective refraction index is lower than zero [38].

**Fig 2:**
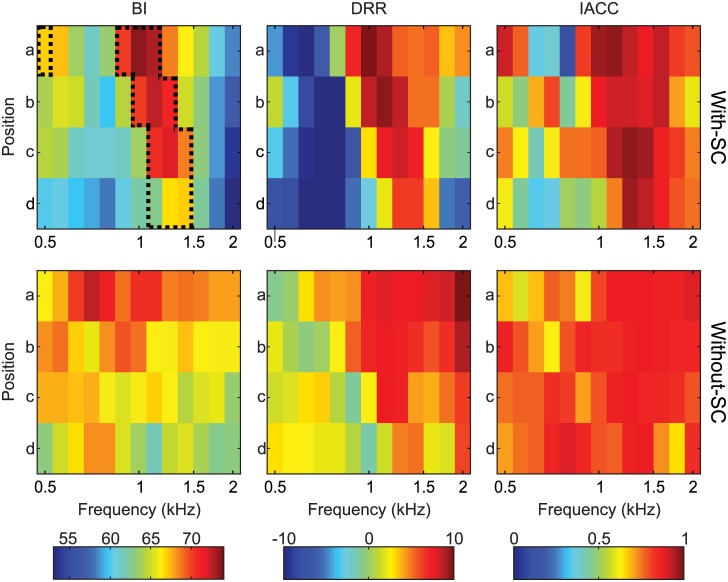
Binaural intensity BI (left), interaural cross-correlation IACC (center) and direct to reverberant ratio DRR (right) for one-third-octave noise bands. The top plots corresponds to the recordings made with the sonic crystal slab between the source and the receiver (condition with-SC) and the bottom plots to the room response (condition without-SC). The results were obtained along the symmetry axis with a perpendicular distance to the slab (a-d). The results for the BI and DRR are expressed in dB, while the IACC is plotted in non-dimensional units. The dashed black frame delimits the focalization region defined in Eq 1.

As we mentioned in the previous section, we selected three acoustical cues that have a proven or likely correlate with the auditory perception of distance: the binaural intensity (BI), the direct-to-reverberant energy ratio (DRR) and the interaural cross-correlation (IACC). The values of these magnitudes (derived from the recordings, as detailed in the Material and Methods section) are displayed in Fig 2 as columns, for the conditions with-SC (top) and without-SC (bottom). Within each plot, the acoustical cues are displayed as a function of the central frequency of the noise bands and the position along the central axis (a-d on Fig 1b). The most noticeable difference introduced by the sonic crystal is an increase of the magnitudes for a central region of the plot, corresponding to the negative-refraction focusing. Also note that the position of the maximum depends on the central frequency. At the beginning of the focusing region (0.89 kHz) the greatest increment occurs at position *a* (0.8 m from the speaker) and as the frequency is increased the maximum is shifted away from the SC, reaching points *c* and *d* at the ending of the focusing region (1.41 kHz).

As we are interested in the possible perceptual effects in the ADP within the focusing region we employ the values of the BI for defining the boundaries of this region in frequency and position. The focusing region, thus, includes the positions and frequencies for which the BI for the with-SC condition is greater than for the without-SC condition. Mathematically:

$$FOC=\{\begin{array}{cc} 1, & \text{if}\;BI_{with-SC}-BI_{without-SC}>0\\ 0, & \text{otherwise} \end{array}$$ (1)

The limits of this region are plotted in the top-left plot of Fig 2 using black dashed lines. The focusing effect is clearly apparent for the BI magnitude, but a relative increase is also observed for the DRR and IACC. For the three acoustical cues, these increases occur roughly at the same distances and frequency bands. In fact, the three cues (BI, DRR and IACC) covariate showing that the focusing is not an intensity-only phenomenom. The measured correlation were: *r* = 0.662 between BI and IACC (*p* < 0.001); *r* = 0.745 between BI and DRR (*p* < 0.001); and *r* = 0.529 between IACC and DRR (*p* < 0.001).

### Psychophysical experiment

Using the same sound samples and experimental configuration as in last section (see Fig 1b), we performed binaural recordings to obtain the stimuli for the psychophysical experiment. Once the stimuli were recorded, four extra stimuli sets were obtained by digitally altering those recordings (see Materials and Methods for a detailed explanation), three of them keeping one of the perceptual cues previously presented, and a last one as a control set, containing none of them. Therefore, the five stimuli set used for the experiment were: (1) the original recordings, where all cues are present (full-cue condition); (2) intensity-only stimuli; (3) DRR-only stimuli; (4) binaural-only stimuli; and (5) control stimuli. For this last type of stimuli there are still spectral differences between the two conditions due to the filtering of the SC slab.

Subjects were presented through headphones with pairs of stimuli that only differed in the presence (with-SC condition) or absence (without-SC condition) of the sonic crystal when they were recorded. For each pair, subjects were asked to judge and report which stimulus was perceived *farther*. We defined the case in which a noise band recorded in the *absence* of the sonic crystal was perceived *farther* than the same band recorded in the presence of the sonic crystal as a case of *positive shift* in the auditory perception of distance. An illusion of proximity of the source will be observed when statistical significant positive shifts occur.

The experimental subjects were divided in two groups: group A (n = 4) where subjects used individualized binaural recordings to generate their stimuli sets and group B (n = 19), where subjects selected a binaural recording set from the ones previously recorded for group A to generate their stimuli sets. For group A the experiment was performed using the recordings made at the four positions (a-d in Fig 1b), while for group B only the recording positions (a) and (c) were employed in order to complete the whole experiment in a single session (see Materials and Methods for a detailed explanation of the recording and selection protocol).

We computed the empirical probabilities as the relative frequency of positive shifts across subjects over all trials for each noise band, position and stimuli set for both groups (see S1 Fig for details). Next, we computed confidence intervals and p-values associated to the null hyphotesis that *the empirical probabilities are not statistically significant different from 0.5* (chance). This allowed us to classify the response into three cases (positive shift, negative shift and no shift) according to the bounds of the confidence intervals. If the upper bound of the confidence interval is below 0.5, we say that the source was consistently perceived farther for the with-SC condition and hence, a negative shift occurred. On the contrary, if the lower bound of the confidence interval is greater than 0.5, we say that the source was consistently perceived farther for the without-SC condition, and hence a positive shift occurred. Finally, if the confidence interval includes the 0.5 value, we say that the source was not consistently perceived farther for any condition, and hence no shift occurred.

**Fig 3:**
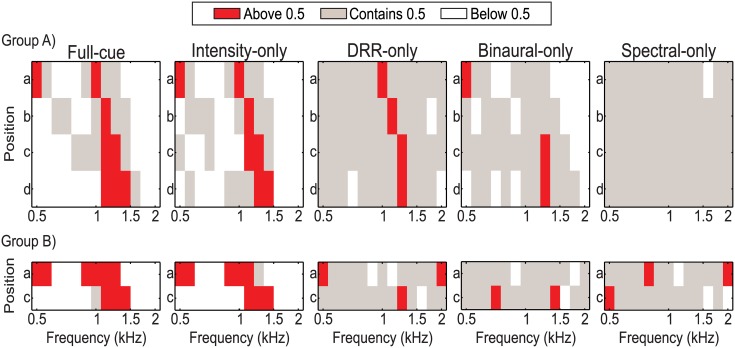
Positive shifts for each stimuli set (1–5) as a function of frequency and position. Proportion of positive shifts classified into three cases according to their confidence limits (*α* = 0.05) as a function of frequency, position and stimuli set for group A (top) and group B (bottom). The white region corresponds to statistically significant negative shifts; the gray region corresponds to ambiguous cases where no shift occurred; and the red region corresponds to statistically significant positive shifts, meaning that the source is consistently perceived farther for the without-SC condition.

In Fig 3 we show the result of this analysis as a function of frequency, position and stimuli set, for group A (top) and B (bottom). This classification leads to a pattern consisting in a majority of positive shifts occurring in the same regions where the acoustical cues are higher (focusing region), for stimuli sets 1–4 in group A and 1–2 for group B. For stimuli sets 1–2 in both groups we can also see a systematic negative shifts for the regions not belonging to the focusing region. Stimuli set 5 on group A and 3–5 on group B give rise to mostly ambiguous cases.

**Fig 4:**
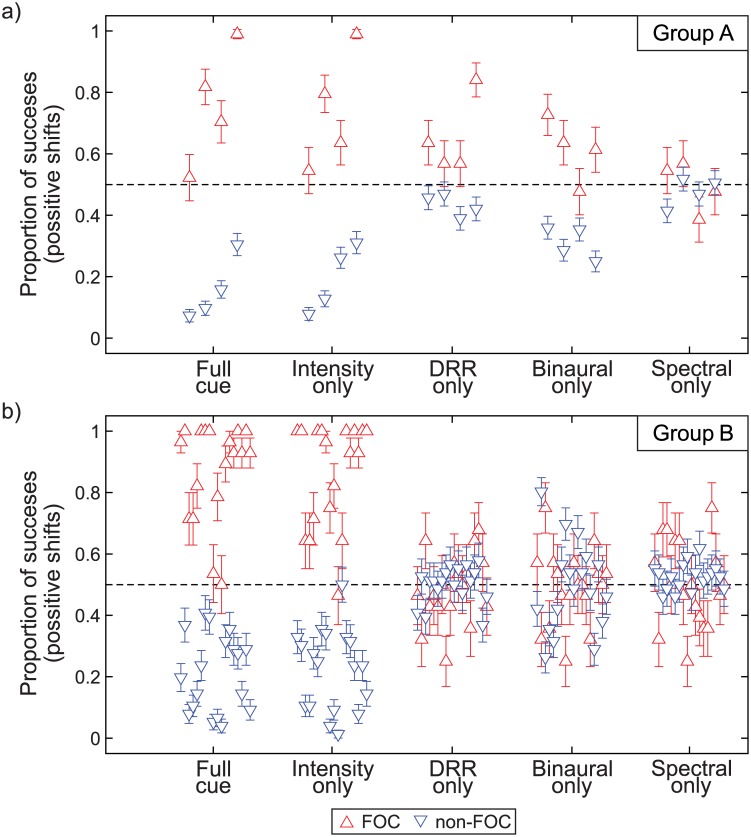
Effect of *focusing* on the response. Individual mean and standard errors of the effect of *focusing* on the response, separated by factors focusing and stimuli set for **(a)** group A and **(b)** group B. Responses under (non-) FOC refers to stimuli (not) belonging to the focusing region defined in Eq 1. The central dashed line indicates the chance level of the response.

In order to verify the relation between the focusing region and the positive shift, we computed the individual mean response for each stimuli set, subject and focusing condition, collapsing position and frequency factors into the new factor *focusing* according to the region defined in Eq 1. In this way, we can compare the responses for stimuli belonging (FOC) or not belonging (non-FOC) to the focusing region. The individual responses with their standard errors separated by factors focusing and stimuli set are shown in Fig 4 for (a) group A and (b) group B.

**Fig 5:**
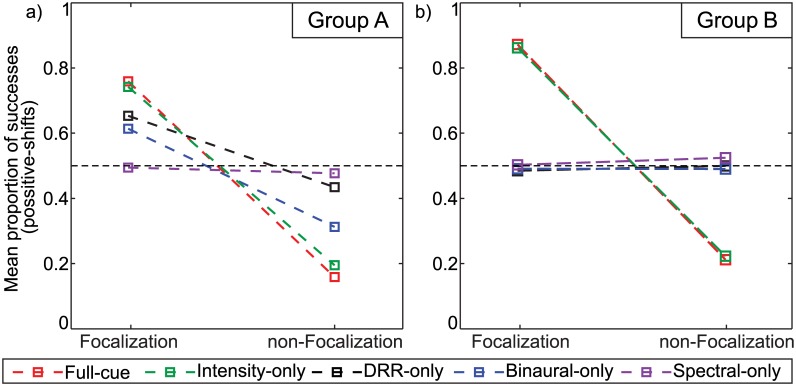
Means of the effect of *focusing* on the response. Means of the effect of the factor *focusing* across subjects separated by stimuli set for **(a)** group A and **(b)** group B.

A whithin-subjects two-way ANOVA showed that the means for different focusing conditions were statistically different both for group A (*F*(1, 3) = 61.8, *p* = 0.004) and group B (*F*(1, 17) = 81.2, *p* < 0.001) and there was no statistically significant effect of main factor stimuli set neither for group A (*F*(4, 12) = 0.62, *p* = 0.53) nor group B (*F*(4, 68) = 1.46, *p* = 0.22). However, there was a statistically significant interaction between stimuli set and focusing for both group A (*F*(4, 12) = 20.5, *p* < 0.001) and group B (*F*(4, 68) = 125, *p* < 0.001), indicating that the differences on the means between the FOC and non-FOC conditions were dependent on the stimuli set. Means of the effect of *focusing* across subjects separated by stimuli set are shown in Fig 5 for (a) group A and (b) group B.

We tested this dependence using one-tailed t-tests between the means corresponding to the same level of factor stimuli set, the null hypothesis being that the mean response for the FOC condition was not higher than for the non-FOC condition. Results are displayed in Table 1. Statistically significant differences were found for stimuli sets 1, 2 and 4 for group A and 1 and 2 for group B. These results imply that there is a preponderant influence of the intensity cue on the elicited auditory distance shift. Binaural content plays a role only for the group A, using individualized binaural recordings. On the other hand, stimuli set 3 (DRR-only) showed only a marginal significant result for group A and no effect for group B. Finally, stimuli set 5 showed no differences between the means for both groups, indicating that the spectral content alone cannot account for the reported auditory distance shift.

**Table 1:** Comparisons between means corresponding to the same level of factor stimuli set for both groups. The one-tailed t-tests gave statistically significant differences for stimuli sets 1 and 2 (both groups), and 4 (group A only), showing the preponderant influence of the BI. Stimuli set 3 (DRR-only) showed only a marginal significant result for group A. The asterisks indicate differences that were significant at the 0.05 level.

	Group A		Group B	
Stimuli set	p	*α*_*HB*_	p	*α*_*HB*_
1 (Full-cue)	0.0013	0.0100*	1.3e-15	0.0100*
2 (Intensity-only)	0.0027	0.0125*	1.4e-11	0.0125*
3 (DRR-only)	0.0251	0.0250	0.65	0.0250
4 (Binaural-only)	0.0067	0.0167*	0.99	0.0500
5 (Spectral-only)	0.3528	0.0500	0.59	0.0167

In order to further investigate the association between the three different selected perceptual cues and the positive-shifts in the perceived distance to the source, we performed a correlational analysis between the experiment under the different conditions. In Table 2 we display correlation coefficients between the probabilities of success (an occurrence of a positive shift) for stimuli set 1 (full-cue) and the probabilities obtained under the other four conditions, along with their confidence intervals. Confidence intervals above 0.5 and highly significant (*p* < 0.001) positive correlations were found for stimuli set 2 (Intensity-only) for both groups. For group A, significant positive correlations (*p* < 0.05) were also found for stimuli sets 3 and 4. The remaining cases showed no significant correlations. These results indicate that the most influential clue in the ocurrence of positive-shifts was the intensity. Binaural and DRR cues have influence only for the group using individualized binaural recordings. On the other hand, spectral changes are insufficient to elicit a change in the localization of the source for both groups.

**Table 2:** Measure of correlation between probabilities. Pearson’s product-moment correlation coefficients *r* between the probabilities of success for stimuli set 1 (full-cue condition) and the probabilities obtained under the other four conditions, along with their confidence intervals. The confidence intervals (CI) are given when the *r* values are significantly different from chance (*p* < 0.05).

	Group A		Group B	
Stimuli set	*r*	CI	*r*	CI
2 (Intensity-only)	0.92	0.87/0.96	0.987	0.971/0.994
3 (DRR-only)	0.44	0.19/0.64	-0.046	-
4 (Binaural-only)	0.79	0.67/0.88	0.14	-
5 (Spectral-only)	0.09	-	-0.20	-

## Discussion

### How the increase of the different cues of ADP could explain the illusion of proximity of the source?

The results reported here show that the presence of a sonic crystal between an acoustic source and a listener creates an illusion of proximity (a significant reduction of the perceived auditory distance), and that this occurs for a certain range of frequencies corresponding to the focusing phenomenon.

The analysis of the differences in the acoustic field for the with- and without-SC conditions indicates that this effect could be explained by the increase in three acoustical cues related to ADP: binaural intensity, direct-to-reverberant energy ratio and interaural cross-correlation.

#### Binaural intensity

The most noticeable acoustical effect generated by the SC is an increase in the binaural intensity, which is strongly dependent on the frequency of the auditory stimulus and, in a lesser degree, on the listener position (see Fig 3). As it was previously reported, the negative refraction generates a virtual source from a convergent wavefront [21, 23]. The increase in intensity thus emerges as a consequence of the energy focusing at that point. As a result of the changes in the negative refraction index with the frequency, the position of the maximum increase displaces over the central line (see Fig 2, top left). Moreover, as the negative refraction also depends on the incident angle and we worked with a nearly cylindrical sound source, the focusing image is elongated in direction perpendicular to the SC, giving rise to the stretched focusing region, as displayed in Fig 1b.

The results obtained in the psychophysical experiment clearly indicate that this increase in BI is the most effective cue for inducing the illusion of the proximity of the source within the focusing region (see Fig 3 and S1 Table in Supporting Information).

The response obtained in the presence of the SC for stimuli set 1 (full-cue condition) can be explained almost entirely by the significant increase of BI induced when the SC slab is placed between the sound source and the listener. This result was appreciated as a highly significant correlation between the experimental responses under full-cue and BI conditions (*r* = 0.92 for group A and *r* = 0.987 for group B, see Table 2).

As we have seen, when the stimulus belongs to the focusing region, the sonic crystal produces a strong increase in the sound intensity and therefore an illusion of proximity of the source. On the contrary, when the stimulus frequency is outside from the focusing region, the sonic crystal induces a strong decrease in the intensity and thus the source is perceived farther. This double outcome of the sonic crystal is reflected as a highly significant effect of the focusing factor (*p* = 0.0027 and *p* = 10^−11^ for group A and B respectively) which makes the sonic crystal a very interesting tool that allows us to induce strong distance shifts by simply changing the frequency of the auditory stimulus.

The strength of this result can be observed for the two groups tested and therefore is independent of the use of individualized or non-individualized binaural recordings. This result is consistent with previous results indicating that the intensity is the most influential cue for ADP, that is available in all listening situations and for all audible sources, regardless of the distance from the listener [29, 30, 32].

#### Direct-to-reverberant energy ratio

Along with the increase in the BI, the presence of the sonic crystal slab induces an increment of another cue for auditory distance perception: the direct-to-reverberant energy ratio. This increment in DRR can be understood by examining how the contributions of the direct sound and the reverberant field change when the sonic crystal is placed between the source and the listener. From the energy values of these two contributions calculated separately (see Materials and Methods) we observe that: (a) the sonic crystal induces an energy increase of the reverberant part of the impulse response of 6–8 dB for all frequencies; (b) the changes in the energy of the direct sound when the sonic crystal is present are strongly frequency-dependent, being more negative in dB for the band-gap and rising up to 10 dB for the focusing range. Therefore, within the frequency range corresponding to the focusing phenomenon, there is an overall increase of the DRR of about 2 dB.

The increase of the reverberant energy induced by the sonic crystal can be explained from two factors. One is the diffraction of the wavefronts that occurs for all frequencies studied, that contributes to the spatial spreading of the energy of the direct sound and the first reflections. As a consequence, early reflections are nearly absent and their energy is allocated to the reverberant field. The other factor is the temporal spreading induced by the sonic crystal (the effective group velocity is reduced for certain frequency bands) [39] that contributes to transfer the energy from the direct sound to the reverberant part of the response. On the other hand, the increase of the energy of the direct sound within the focusing range can also be explained from the virtual source formed by the convergent waveforms. The energy that emerges from the source at slightly divergent angles, which would contributed to the early reflections, is concentrated in the focusing region and contributes to the direct sound.

The influence of the changes in DRR values on the perceived distance to the source is also observed in our experiment. However, it is worth noting that this effect was marginal and it was observed only for the group A, where individualized binaural recordings were used. For group A (see Fig 3), increased DRR values induced by the SC in the focusing region were sufficient to induce an illusion of proximity of the source (see S1 Table in Supporting Information). By contrast, in the non-focusing region, decrease in DRR values caused an significative increase in the perceived distance from the source. Again, although with a smaller effect than that seen in BI condition, this induces significant difference in the effect of the focusing factor (*p* = 0.0251). The fact that changes caused by the sonic crystal induce changes in the perceived distance of the sound source is consistent with numerous previous studies showing that the DRR is an influential absolute auditory cue for ADP [28–30, 33, 40]. However, for participants of group B the increase in the DRR induced by the presence of the sonic crystal was not sufficient to induce a significant change in the distance from the source both in the FOC and non-FOC region (see S1 Table in Supporting Information). Moreover, within this group no significant differences in the effect of the focusing factor (*p* = 0.65) were observed. This can also be observed in the significant decrease (from 0.92 for Intensity-only vs. 0.44 for DRR-only condition) of the correlation coefficients (see Table 2).

The marginal effect obtained here with DRR, compared with that obtained in intensity-only condition, agrees with a recent study by Kolarik et al [41] which investigates how listeners used sound intensity level and DRR for discriminating ADP. The authors showed that performance on the basis of sound intensity level was generally better than performance based on DRR only. Also, they showed that this last performance is significantly improved when the reverberation is increased, until DRR and level provided equally effective information for highly reverberant environments. Based on th is, we consider that the relatively short reverberation times for the frequency bands where the effect of the SC slab was more noticeable could partly explain the weak effect of DRR on the illusion of proximity of the sound source.

Another possibility, which does not exclude the above, is that the fact of having excluded the binaural information from DRR-only stimuli has affected the correct perception of DRR. Pioneering studies of ADP consider DRR as a monaural cue. However, there is some controversy regarding the mechanisms involved in the separation between direct and reverberant field of a sound arriving to the listener’s ears. Although several authors suggested various criteria for calculating the DRR and several time windows have been proposed to differentiate the direct sound of the reverberant, no generally accepted standard has emerged to date [32]. Moreover, works by Bronkhorst [34, 42] posit another approach and consider that reverberation contains binaural information from the sound source that listeners use to estimate its distance. Bronkhorst proposes a model in which interaural time differences instead of arrival time are used to determine which reflections should be added to the direct sound. This hypothesis suggests that the auditory system could potentially use directional information to separate direct and reverberant sound [24]. Therefore, the fact that we eliminated the binaural information in the DRR-only condition may have affected the correct perception of this cue and then the correct perception of distance from the sound source.

#### Interaural cross-correlation

The increase in the IACC that also occurs in the focusing region can be partially explained by the increment in the DRR, since the two magnitudes are related [37]. In fact, a simple model can be made in order to show this dependence. We can consider the overall binaural signal as a sum of two contributions: a diotic signal corresponding to the direct sound with *IACC* = 1 (maximum correlation) and a dichotic signal corresponding to the reverberant part of the sound with a residual IACC value. This residual cross-correlation value arises because the reverberant field is not completely incoherent and can be obtained after passing the direct sound through a reverberation model [43].

Combining these two signals (perfectly correlated direct sound and poorly correlated reverberant sound) with different amplitude ratios, we can reproduce the curve obtained by Larsen et al (see Fig. 1 in [37]), showing the dependence of the IACC with the DRR. Comparing this theoretical curve with our data we obtain a good agreement for the recordings made without the SC. However for the case of the recordings made with the SC, the IACC obtained are systematically higher than those predicted by the curve (see S2 Fig in Supporting Information). Statistical analysis revealed that the null hypothesis of the residuals possessing a normal distribution with zero mean could not be rejected for the first case (*p* = 0.48), while for the case with the SC the same hypothesis can be rejected highly significantly (*p* < 10^−12^). Therefore the increase in the IACC is probably related to another effect that it is not included in the DRR. One interesting possibility is that the convergent wavefronts formed by the focusing phenomenon could be less scattered by the head and torso than the wavefronts from the direct sound. Thus the virtual source (convergent wavefront) would have a greater IACC than the real one (divergent wavefront).

As in DRR, the increase in IACC values induced by the sonic crystal is reflected as an illusion of proximity of the sound source only for the group that performed the experiment with individualized binaural recordings (group A). In fact, for this group the illusion was stronger for the IACC-only condition than for the DRR-only condition (see Fig 4). This is clearly noticeable when comparing the effect of the focusing factor for IACC-only and DRR-only conditions (*p* = 0.0067 vs. *p* = 0.0251 respectively, see Table 1) and it is also reflected in the values of correlation coefficients between probabilities (*r* = 0.77 vs. *r* = 0.44 respectively, see Table 1 and Fig 3). The results obtained with individualized binaural recordings give support to the hypothesis that the reverberation is a binaural ADP cue, and agree with the results reported by Bronkhorst [34] where a larger IACC caused a smaller perceived distance from a virtual sound source. However, similarly to what was observed in DRR-only condition, when non-individualized binaural recordings were used (group B, see Fig 3), changes in the IACC caused by SC were not sufficient to induce significant changes in the perceived distance of the sound source. Moreover, in this group no significant differences in the effect of the focusing factor (*p* = 0.99) were observed.

It is worth noting that the changes in IACC resulted in a significant change in the perceived distance to the source only when individualized binaural recordings were used (group A). What caused this difference? Although from the results obtained in this work we cannot give a conclusive answer to this question, possible explanations could be related to the lower degree of externalization when using non-individualized binaural recordings. Externalization is achieved when the sound sources are perceived outside the head even when the acoustical signal is delivered through headphones, and is a necessary condition for the correct estimation of distance in virtual acoustic environments [26, 44]. There are a considerable number of works that addressed this issue for different conditions. Modern consensus is that the higher the degree of realism of the signal delivered through headphones, the more likely that the subjects externalize the sound sources [44]. Among the signal characteristics that must be preseved for achieving a higher degree of realism are: the low-frequency interaural phase differences [45], the temporal modulation of the interaural level differences [46], and the spectral detail (including the cues provided by the pinna filtering) [47]. Another important factor that enhances the externalization is the reverberation (even to the point that when this factor is present other factors have little or no influence [48, 49].) However, for the binaural-only stimuli set the reverberation tail was removed and the subjects are forced to judge the distance to the source relying only on spectral and binaural information. Hence, it is possible that subjects from group B, using non-individualized binaural recordings, were less likely to achieve a high degree of externalization, being less sensitive to the effects of the binaural information on the distance to the source. Nevertheless, we believe that is necessary to address future rigorous studies to test this hypothesis and the role of the IACC in ADP.

### Can the illusion be predicted from previously reported difference limens for the ADP cues?

We are now interested in investigating whether the observed positive shifts can be explained from the differences in the selected acoustical cues between the conditions with-SC and without-SC. Our aim is to determine if these positive shifts can be predicted from the difference limens reported in the literature for the three acoustical cues. To this end, we first establish which values of position and frequency in our experiment lead to differences between the condition with-SC and without-SC above the difference limen of some acoustical magnitude (BI, DRR or IACC), and then investigate if there is a significant correlation with the positive shifts reported by the subjects.

The perceptual correlate of the binaural intensity is the binaural loudness [50]. The difference limen for the loudness for filtered noise depends on the duration, central frequency, bandwidth and intensity of the stimulus. However, narrowband filtered noise also have an inherent variability that must be taken into account. In our case, since the signals delivered to the loudspeakers for the conditions with- and without-SC were the same, we were restricted to the case of ‘frozen’ noise. Buus and Buus and Florentine [51, 52] studied the difference limens for narrowband filtered frozen noise, but presented monoaurally and for stimulus duration much shorter than those used here. Majernik [53] determined the difference limens for similar stimuli while extending their durations up to 400 ms. Under the hypothesis that incorporating binaural information and increasing the duration will reduce the difference limen [54], the value of 1.38 dB for the difference limen of one critical band centered around 1 kHz presented monoaurally at a level 60 dB and lasting for 400 ms, obtained in the previously cited work, can be considered an upper bound for the difference limens in our experiment. The chosen stimulus was the most similar to ours. Yet, it is also useful to derive a better estimate of the difference limen for the binaural loudness of narrowband filtered frozen noise. To this end, we calculated the binaural loudness for all our stimuli and for the long duration stimuli of Majernik using the method described in [50]. We then adjusted the probability of discrimination for the published data, as a function of the binaural loudness, using:

$$P=\frac{1}{1-\exp(L_{b}-L_{b0})/\sigma }$$ (2)

where *L*_*b*_ (*L*_*b*0_) is the binaural loudness for the signal (reference) condition and *σ* = 0.16 sones. In our case the signal (reference) condition correspond to the stimuli with (without) the sonic crystal.

There are several works that report just noticeable differences (JND) for the direct-to-reverberant energy ratio, giving partly contradictory results [37, 41, 55, 56]. Zahorik reports a JND approximately constant of 5 to 6 dB for four type of stimuli, presented medially and laterally. Larsen et al., spanning a larger range of DRR values, found that the JND has an *U* shape with a minimum value of 2.4 dB for DRR = 0 dB and rising to 3 dB and 8 dB for positive DRR values of 10dB and 20 dB, and to 6 dB for DRR = -10dB. Akeroyd derived psychometric curves for the discrimination of distance under normal conditions (where intensity is the primary cue) and for equalized level conditions (where DRR and binaural cues are present). Following a similar method, Kolarik obtained psychometric curves for different reverberation conditions attaining somewhat lower discrimination thresholds. A clear finding from these last two works is that the JND decreases along with an increase of the reverberation. Comparing the results for the DL of DRR derived by Kolarik [41] and by Akeroyd [56], from experiments on discrimination of distance, to those obtained by Larsen et al. [37] from experiments on discrimination of reverberation it appears that these last experiments give much lower discrimination thresholds. Moreover, the reduction of the difference limens when the DRR decreases is not observed, or even reversed. One possible explanation is that this reduction is caused by changes in the binaural cues for distance that are not present in the experiment performed by Larsen et al. Another result obtained by these last authors that points in this direction is that there are no significant differences between the discrimination thresholds when the stimuli are presented monaurally and binaurally, indicating that binaural information is not taken into account for the discrimination of reverberation. Besides these particular differences, a general account of the influence of the different cues for ADP could be as follows. When there are level differences between the stimuli this is the dominant cue. For level-equalized stimuli with DRR values close to 0 dB this is the most important cue, and focusing on this feature (for example in a targeted experiment) reduces the discrimination threshold. When the DRR values are negative (higher reverberation) this cue is impoverished but the binaural cues are relevant, for this reason the thresholds for the experiments by Akeroyd and Kolarik decrease while for the experiment by Larsen et al. rises (the subjects are not taking binaural information into account). Finally, for large positive values of DRR (lower reverberation) both cues are weak and the discrimination thresholds increase for all experiments.

In our case, since we are discriminating the energetic component of the DRR (after a phase randomization) the discrimination thresholds obtained by Larsen are more adequate as a reference. We adjusted sigmoidal psychometric curves for the probability of discrimination as a function of DRR, using interpolated values for the thresholds as a function of the pedestal DRR value (see Fig. 3 in [37]).

Lastly, we derived psychometric curves for the probability of discrimination as a function of the IACC value from the discriminable changes in cross-correlation reported by Pollack and Trittipoe [57], also using sigmoidal functions.

In this way we obtained, for the stimuli sets intensity-only, DRR-only, and binaural-only in our experiment, the probability of discrimination between the conditions with- and without-SC, adjusting previously reported results. The correlation coefficients between these probabilities and the number of times that subjects reported a positive shift for the intensity-only stimuli set were r = 0.85 (*p* < 0.001) for the group A, and r = 0.89 (*p* < 0.001) for group B. For the other two stimuli sets statistically significant correlations were found only for group A: r = 0.54 (*p* < 0.001) for the DRR-only, and r = 0.6 (*p* < 0.001) for the binaural-only condition. These results indicate that, given the changes in intensity, DRR and IACC induced by the sonic crystal, when a significant (or marginal) effect of the focalization in the auditory distance is observed, the possitive shifts on the perceived distance can be explained by the discrimination thresholds reported in the literature for these auditory distance cues.

## Materials and Methods

### Recordings

#### Room

Binaural recordings were conducted in a rectangular room, approximately 12 × 7 × 3 m (length × width × height) with walls covered by sound-absorbing panels (pyramid polyurethane acoustical foam, 50 mm), the floor by carpet, and the ceiling by fiber glass acoustic roof panels. Additional sound-absorbing panels (pyramid polyurethane acoustical foam, 35 mm) mounted on vertical plates (dimensions 7.5 m width and 2.5 m height) were placed 1.5 m from the right (according to the subject’s point of view) wall in order to cut the reflections from a concrete counter (see Fig 1a). As a consequence, a slight asymmetry on the measured sound intensity field is observed (see Fig 1b). The sonic crystal slab and loudspeaker were centered between the left wall and the vertical plates. The room has an average reverberation time (T30, A-weigthed) of 0.45 s, and the background noise level during the recordings and the psychophysical experiment was approximately 19 dBA. We have chosen a room with a small but noticeable reverberation because the direct-to-reverberant energy ratio was used as a cue for ADP during the experiments.

#### Sonic Crystal

The two-dimensional SC slab used for the recordings was built using 59 PVC cylinders of 2 m height and 16 cm diameter, arranged in a triangular lattice configuration, with lattice parameter of 22 cm. The first partial band gap (for normal incidence) extends approximately from 0.57 to 1 kHz, while the first negative-refraction focusing band is in the range between 1 and 1.5 kHz, calculated using the plane-wave method (see band diagram on Fig 1b).

#### Sample recording

The sound samples used for the recordings consisted of thirteen one-third-octave noise bands with central frequency ranging from 0.5 to 2 kHz and one-sixth-octave steps. Sound samples were prepared digitally using Matlab (Mathworks Inc.) at 96 kHz sampling frequency and 16-bit sample depth, and reproduced via a Genelec 8030 two-way loudspeaker unit connected to a MOTU 896mk3 digital audio interface. The duration of all samples were two seconds with onset and offset rounded by a raised-cosine function of 50 ms. Binaural recordings were made using a custom designed binaural dummy head equipped with SP-TFB-2 intra-aural microphones connected to a Tascam DR40 hand-held recorder, with 96 kHz sampling frequency and 16-bit sample depth. The sound source was located 0.42 m from one side of the SC slab (0.92 m width) and the recording points were located at 0.8, 1.8, 2.8 and 3.8 m from the other side of the SC.

### Definition of acoustical cues

#### Binaural Intensity (BI)

We calculated the binaural intensity (BI) from the recordings by linearly adding the intensity of the signal reaching both ears as follows:

$$\begin{array}{ccc} I_{l,r} & = & \frac{1}{L}\int_{0}^{L}p_{l,r}^{2}\left(t\right)dt \end{array}$$ (3)

$$\begin{array}{ccc} BI & = & 10\log_{10}\left[{\left(I_{l}I_{r}\right)}^{1/2}/I_{ref}\right] \end{array}$$ (4)

where *I*_*l*,*r*_ stands respectively for the left and right ear individual intensity, *p*_*l*,*r*_(*t*) for the A-weighted pressure field on each ear, L for the length of the recorded signal, and *I*_*ref*_ = 10^-12^W/m^2^ is the reference intensity.

#### Direct to Reverberant Energy Ratio (DRR)

We obtained a set of binaural impulse responses for each condition and position of the sound source using the exponential sweep technique described in [58]. Next, the DRR for each frequency band was calculated by filtering the impulse response with the same one-third-octave filters used for the generation of the samples. In order to separate the direct sound from the reverberant field in the impulse response we used the same weighting function as proposed by Bronkhorst and Houtgast [33]:

$$w\left(t\right)=\{\begin{array}{cc} 1, & \text{for}\;0<t\leq t_{w}-\frac{\pi }{2s}\\ 0.5-0.5\sin[s\left(t-t_{w}\right)] & \text{for}\;t_{w}-\frac{\pi }{2s}<t<t_{w}+\frac{\pi }{2s}\\ 0, & \text{for}t\geq t_{w}+\frac{\pi }{2s} \end{array}$$ (5)

where *t* = 0 is the time of arrival of the direct sound, and the duration *t*_*w*_ and slope *s* were adjusted to 7 and 2 ms respectively, in order to include the direct sound and the first reflection from the floor. This early reflection was included in the time window because it was either very close to the direct sound in absence of the SC (with a delay between 2 and 5 ms), or completely fused with the direct sound when the SC was present. Using this weighting function *w*(*t*) we calculated the *DRR*_*n*_ for the *nth* frequency band through:

$$\begin{array}{ccc} D_{n} & = & \int h_{n}^{2}\left(t\right)w\left(t\right)dt \end{array}$$ (6)

$$\begin{array}{ccc} R_{n} & = & \int h_{n}^{2}\left(t\right)\left(1-w\left(t\right)\right)dt \end{array}$$ (7)

$$\begin{array}{ccc} DRR_{n} & = & \log_{10}(\frac{D_{n}}{R_{n}}) \end{array}$$ (8)

where *h*_*n*_(*t*) is the impulse response corresponding to the nth band filtered noise, and *D*_*n*_ (*R*_*n*_) is the energy of the direct (reverberant) sound.

Finally, we obtained a single value of *DRR*_*n*_ for each position, condition and frequency band by averaging the *DRR*_*n*_ calculated for both ears.

#### Interaural Cross Correlation (IACC)

The IACC is the maximum value of the interaural cross correlation function IACF(*τ*) as a function of the time difference between ears (*τ*) [59]:

$$\begin{array}{ccc} \phi_{lr}\left(\tau \right) & = & \frac{1}{2L}\int_{-L}^{+L}p_{l}\left(t\right)p_{r}\left(t+\tau \right)dt \end{array}$$ (9)

$$\begin{array}{ccc} IACF(\tau ) & = & \phi_{lr}\left(\tau \right)/{\left[I_{r}I_{l}\right]}^{1/2} \end{array}$$ (10)

Since our recordings were made along the symmetry axis it is expected that the maximum of the IACF would always occur for *τ* values very close to zero.

### Psychophysical experiment

#### Subjects

Group A (using individualized binaural recordings) was composed by four male subjects (n = 4), with ages ranging between 30 and 41 y.o. and group B (using non-individualized binaural recordings) was composed by sixteen male subjects and three female subjects (n = 19) with ages ranging between 23 and 53 y.o. All the experimental subjects reported normal hearing condition. Two subjects from group A and fifteen subjects from group B had also musical expertise.

#### Ethics Statement

The experiments were undertaken with the understanding and written consent of each subject, following the Code of Ethics of the World Medical Association (Declaration of Helsinki) and were approved by the Ethics Committee of the National University of Quilmes.

#### Procedure

The experiment was divided into five blocks, one for each stimuli set. The first block always corresponded to stimulus set 1 while the remaining blocks were performed in random order. Each block consisted in the repeated presentation of a pair of stimuli corresponding to the same filtered noise band and position, but differing with respect to the presence (with-SC condition) or absence (without-SC condition) of the sonic crystal. The stimuli were obtained from the binaural recordings of the thirteen noise bands at the four positions (a-d) for group A and at positions (a) and (c) for group B. The trials were presented in random order, and within each trial the order of the stimuli was also randomized.

The task at each trial was to judge which stimulus was perceived *farther*. Subjects were allowed to play again the stimuli pair as many times as necessary before giving an answer. The choice was forced between the two stimuli and no feedback was provided. Each pair appeared four times, giving for group A (B) a total of 208 (104) test trials per block and a grand total of 1040 (520) test trials for the complete experiment. Each block took approximately 40 minutes for group A and 20 minutes for group B. The five blocks were distributed into two sessions only for group A. Within session, blocks were separated by pauses 15–20 minutes long, in order to minimize fatigue. The protocol was conducted by means of a Matlab script using the Psychotoolbox (version 3) extension.

Subjects from group A were seated in the same room as used for the binaural recordings and received the stimuli through Sennheiser HD-600 open headphones connected to a Focusrite Saffire LE soundcard. We divided group B into two subgroups: for ten subjects we used the same hardware and room as for group A, while for the remaining nine subjects the experiment was conducted in a different room (middle size room with acoustic treatment) using Senheiser HDA200 closed dynamic headphones connected to a Focusrite Scarlett 8i6 soundcard to deliver the stimuli. Subjects were seated in a comfortable position, in front of the computer screen and indicated their responses using the computer keyboard. The screen was used to inform the subject when to report his/her judgement and to indicate the end of a block. The intensity level was adjusted to a comfortable level for each subject.

#### Stimuli sets

Binaural recordings were obtained by the same methodology as used for the dummy-head samples, but inserting the microphones at the end of the earâ??s canal of the subjects from group A. Next, these recordings were altered using three processes: (a) phase randomization; (b) removal of the reverberation buildup and tail; and (c) loudness normalization. For the first alteration, phase was randomized using a short-time Fourier transform (STFT) resynthesis algorithm in order to keep the temporal envelope of the signal. This algorithm consisted in calculating the STFT, randomizing the phase of each temporal window and finally applying an overlap-add resynthesis method. The removal of the reverberation buildup and tail was simply performed by applying a rounded rectangular window to the signal. Finally, the loudness normalization was achieved by calculating the binaural loudness of the signal, using the procedure detailed in [50], and adjusting the amplitude of the stimuli until all computed binaural loudness differences were less than 0.001 sones. Five stimuli sets were then obtained combining these modifications: (1) the original recordings where all the perceptual cues are present (full-cue condition); (2) Intensity-only stimuli, after modifications (a) and (b); (3) DRR-only stimuli, after modifications (a) and (c); (4) Binaural-only stimuli, after modifications (b) and (c); and (5) control stimuli, after modifications (a), (b) and (c).

Subjects in group A used their individual binaural recordings while subjects in group B had to select a binaural recordings set from the obtained for subjects in group A. This selection was made by performing a pre-test that allowed us to determine the best suited binaural recording set for each subject, based on the degree of externalization achieved. The experiment consisted in a rating task, similar to the one proposed by Catic et *al.* [46], where subjects were instructed to rate the degree of externalization using four levels (The options were presented in spanish) “The sound is perceived:” (1) “inside the head”, (2) “very close to the head”, (3) “outside the head but non-localized”, and (4) “outside the head and localized”. The stimuli used were seven of the filtered noise bands prevoiusly recorded (0.89 to 1.78 kHz) at position c and belonging to the without-SC condition. Each noise band was repeated three times giving a total of 105 trials. The binaural recording set was selected based on the mean score of externalization rated by the subjects and used for all the experimental procedure.

#### Statistical Analysis

Binomial statistics was used to calculate the probability of positive shifts (stimulus without-SC perceived farther) for each subject, position, frequency and stimuli set where success was identified with a positive shift. Confidence interval at the 95% level were calculated using the Clopper-Pearson formula after pooling all the trials of all subjects. Associated p-values were calculated against the null hyphotesis **H*_0_: the true probability of a positive-shift is 0.5* by means of a (two-tailed) exact binomial test.

Individual mean responses were then recalculated as a function of stimuli set and focusing condition, as defined in Eq 1. The effect of these two factors on the perception of positive shifts was analyzed by means of a two-way within-subjects ANOVA. The Huynh-Feldt correction was used when the condition of sphericity was not satisfied. The interaction effect between factors was analyzed by means of one-tailed paired t-tests between the means corresponding to the same level of factor stimuli set (**H*_0_: the probability of a positive shift was higher for the FOC condition*). The experiment-wise error was controlled by means of the Bonferroni-Holm correction [60]. All the correlations were measured using the Pearson product-moment correlation coefficient.

Differences among the two subgroups of group B (see Procedure for a description of the groups and subgroups) were assessed by means of two-sample unpaired t-tests on the individual responses. These tests were performed separately for each position, frequency and stimuli set. Using a significance level of 0.05, only six comparisons over 130 indicated statistically significant differences between the two subgroups, a result that can be explained by chance alone. Next, we calculated the differences between the mean proportions of positive shifts for each position, frequency and stimuli set. Considering each stimuli set separately, the differences were distributed around zero, with means ranged between 0.01 and 0.05 (see S3 Fig in Supporting Information). For each stimuli set, a one-sample t-test indicated no statistically significant difference between the mean difference and the zero value. Based on these evaluations, we concluded that no differences existed between the two groups and proceeded to pool their data for the statistical analysis.

## Supporting Information

S1 Fig Positive shifts for each stimuli set (1–5) as a function of frequency and position. Percentage of positive shifts (perceiving the source farther for the without-SC condition) across subjects over all trials as a function of frequency, position and stimuli set for group A (top) and group B (bottom).
(EPS)

S2 Fig Interaural Cross-correlation (IACC) as a function of Direct-to-reverberant energy ratio (DRR). Dependence of the IACC with the DRR as calculated from a theoretical model described in the Discussion (black solid line) and derived from the psychophysical experiments for the conditions without-SC (blue squares) and with-SC (red squares).
(EPS)

S3 Fig Statistical analysis of the differences among the two subgroups of group B. Histograms (bin size = 0.1) of the differences on the mean proportions of positive shifts between the two subgroups of group B for each position and frequency separated stimuli set. Dashed red line indicates the mean over positions and frequencies for each stimuli set.
(EPS)

S1 Table P-values for the tests over the mean response against the null hyphotesis. P-values for the tests over the mean response (empirical probabilities of positive shifts averaged across subjects and trials, for each noise band, position, stimuli and group), against the null hyphotesis *the probabilities are not statistically significant different from 0.5 (chance)*. Values lower than 0.05 indicate the stimuli that provoked a statistically significant shift in the perception of distance, as indicated in Fig 3.
(PDF)
