# Supplementary material for: An Auditory Illusion of Proximity of the Source Induced by Sonic Crystals
Source: PLoS One. 2015 Jul 29;10(7):e0133271. doi: 10.1371/journal.pone.0133271 (PMC4519286; doi:10.1371/journal.pone.0133271)
Supplement: S1 Table — P-values for the tests over the mean response (empirical probabilities of positive shifts averaged across subjects and trials, for each noise band, position, stimuli and group), against the null hyphotesis the probabilities are not statistically significant different from 0.5 (chance). Values lower than 0.05 indicate the stimuli that provoked a statistically significant shift in the perception of distance, as indicated in Fig 3. (PDF) [file pone.0133271.s004.pdf]

Group A

**Pos / Frec**	0.50	0.56	0.63	0.71	0.79	0.89	1.00	1.12	1.26	1.41	1.59	1.78	2.00 kHz
a	0.0005188	0.4545	0.0005188	3.052e-05	3.052e-05	1	0.004181	0.07681	0.4545	0.004181	3.052e-05	3.052e-05	3.052e-05
b	3.052e-05	0.004181	0.07681	0.8036	3.052e-05	3.052e-05	0.07681	0.004181	0.4545	0.2101	3.052e-05	3.052e-05	3.052e-05
c	0.02127	0.0005188	3.052e-05	3.052e-05	0.2101	0.4545	0.07681	3.052e-05	3.052e-05	0.07681	0.0005188	3.052e-05	3.052e-05
d	3.052e-05	0.02127	0.0005188	0.0005188	0.0005188	0.004181	0.02127	0.0005188	0.02127	0.0005188	0.2101	0.0005188	3.052e-05
a	0.004181	0.07681	3.052e-05	3.052e-05	3.052e-05	0.8036	0.0005188	0.07681	1	0.07681	0.004181	3.052e-05	0.0005188
b	0.0005188	0.4545	0.4545	0.07681	3.052e-05	3.052e-05	0.8036	0.02127	0.4545	0.8036	0.02127	0.0005188	3.052e-05
c	0.4545	0.004181	0.004181	0.07681	0.004181	0.0005188	0.004181	0.0005188	3.052e-05	0.2101	0.004181	3.052e-05	0.0005188
d	0.004181	0.4545	0.0005188	3.052e-05	0.0005188	0.07681	1	0.2101	0.004181	0.02127	0.07681	3.052e-05	3.052e-05
a	0.07681	0.8036	0.4545	1	0.8036	0.2101	0.02127	0.8036	0.07681	0.4545	0.2101	0.4545	0.8036
b	1	0.4545	0.8036	0.2101	0.07681	1	0.8036	0.02127	0.8036	1	1	0.02127	0.4545
c	0.8036	0.8036	0.8036	0.4545	0.2101	0.4545	0.4545	0.07681	0.02127	0.4545	0.07681	0.8036	0.2101
d	0.4545	0.4545	0.2101	0.02127	0.4545	0.8036	0.2101	1	0.07681	1	0.8036	0.2101	0.004181
a	0.02127	0.4545	0.2101	0.02127	0.004181	0.07681	0.07681	0.8036	0.4545	0.02127	0.02127	0.0005188	0.004181
b	0.2101	0.02127	0.8036	0.8036	0.2101	0.004181	1	0.4545	1	0.2101	0.0005188	0.0005188	0.004181
c	0.8036	0.8036	0.07681	0.8036	1	0.4545	0.2101	0.4545	0.004181	0.4545	0.2101	0.02127	0.02127
d	0.02127	0.07681	0.07681	0.07681	0.07681	3.052e-05	0.8036	0.2101	0.004181	0.8036	0.4545	0.4545	0.0005188
a	1	0.8036	0.8036	0.2101	0.8036	0.4545	0.4545	0.4545	0.8036	0.07681	0.02127	0.2101	0.07681
b	0.4545	0.2101	0.4545	0.4545	1	0.8036	1	1	0.4545	0.2101	0.8036	0.07681	0.07681
c	0.8036	1	0.8036	0.4545	1	0.8036	1	0.8036	0.8036	1	1	1	0.07681
d	0.4545	0.4545	0.8036	0.8036	0.8036	0.4545	0.4545	0.2101	0.07681	0.8036	0.4545	0.8036	0.4545

Group B

**Pos / Frec**	0.50	0.56	0.63	0.71	0.79	0.89	1.00	1.12	1.26	1.41	1.59	1.78	2.00 kHz
a	5.633e-13	0.0007646	6.417e-14	0	2.96e-11	0.01544	5.045e-09	4.334e-12	0.01544	3.851e-07	4.441e-16	0	0
c	4.369e-05	4.713e-06	1.397e-06	1.484e-05	0.02863	4.369e-05	0.2067	6.217e-15	0	0	5.045e-09	0	0
a	5.633e-13	4.369e-05	6.417e-14	0	6.417e-14	1.484e-05	5.045e-09	5.045e-09	0.1354	4.369e-05	5.633e-13	0	0
c	0.08465	1.397e-06	1.484e-05	0.0001206	4.713e-06	0.01544	0.02863	6.217e-15	0	4.334e-12	4.713e-06	0	0
a	0.02863	0.1354	0.5666	0.08465	0.3019	0.003836	0.5666	0.0007646	0.4222	0.3019	0.3019	0.9088	0.08465
c	0.5666	0.08465	0.1354	1	0.5666	0.5666	0.5666	0.731	0.02863	0.9088	0.007905	0.9088	0.05045
a	0.5666	0.3019	0.1354	0.4222	1	0.02863	0.2067	0.731	0.2067	0.05045	0.5666	0.0001206	0.5666
c	0.9088	0.08465	0.5666	0.007905	0.2067	0.05045	0.4222	1	0.08465	0.05045	0.02863	0.5666	0.08465
a	0.731	0.4222	1	0.1354	0.05045	0.731	0.9088	0.05045	1	0.2067	0.3019	0.3019	1.484e-05
c	0.02863	0.05045	0.2067	0.3019	0.731	0.3019	0.1354	0.9088	0.4222	0.1354	0.05045	0.731	0.1354
